# Non-sister Sri Lankan white-eyes (genus *Zosterops*) are a result of independent colonizations

**DOI:** 10.1371/journal.pone.0181441

**Published:** 2017-08-09

**Authors:** Nelum Wickramasinghe, V. V. Robin, Uma Ramakrishnan, Sushma Reddy, Sampath S. Seneviratne

**Affiliations:** 1 Avian Evolution Node, Department of Zoology and Environment Sciences, Faculty of Science, University of Colombo, Colombo, Sri Lanka; 2 National Center for Biological Sciences, Tata Institute of Fundamental Research, Bangalore, India; 3 Biology Department, Loyola University Chicago, Chicago, Illinois, United States of America; National Cheng Kung University, TAIWAN

## Abstract

Co-occurrence of closely related taxa on islands could be attributed to sympatric speciation or multiple colonization. Sympatric speciation is considered to be rare in small islands, however multiple colonizations are known to be common in both oceanic and continental islands. In this study we investigated the phylogenetic relatedness and means of origin of the two sympatrically co-occurring *Zosterops* white-eyes, the endemic *Zosterops ceylonensis* and its widespread regional congener *Z*. *palpebrosus*, in the island of Sri Lanka. Sri Lanka is a continental island in the Indian continental shelf of the Northern Indian Ocean. Our multivariate morphometric analyses confirmed the phenotypic distinctness of the two species. Maximum Likelihood and Bayesian phylogenetic analyses with ~2000bp from two mitochondrial (ND2 and ND3) and one nuclear (TGF) gene indicated that they are phylogenetically distinct, and not sister to each other. The two subspecies of the peninsula India; *Z*. *p*. *egregius* of Sri Lanka and India and *Z*. *p*. *nilgiriensis* of Western Ghats (India) clustered within the *Z*. *palpebrosus* clade having a common ancestor. In contrast, the divergence of the endemic *Z*. *ceylonensis* appears to be much deeper and is basal to the other *Zosterops* white-eyes. Therefore we conclude that the two *Zosterops* species originated in the island through independent colonizations from different ancestral lineages, and not through island speciation or multiple colonization from the same continental ancestral population. Despite high endemism, Sri Lankan biodiversity is long considered to be a subset of southern India. This study on a speciose group with high dispersal ability and rapid diversification rate provide evidence for the contribution of multiple colonizations in shaping Sri Lanka’s biodiversity. It also highlights the complex biogeographic patterns of the South Asian region, reflected even in highly vagile groups such as birds.

## Introduction

Speciation is the evolutionary process by which biological populations diverge into distinct species through reproductive isolation [1–3]. ‘Allopatric speciation’ occurs when populations are reproductively isolated due to a geographic barrier [4]. Genetic differences get accumulated within the isolated populations overtime and cause these geographically separated populations to become distinct species through reproductive isolation. On the other hand in ‘sympatric speciation’, reproductive isolation is non-geographic and the newly diverged species will share the same geographic location [4, 5]. Even though allopatric speciation is common and widely accepted, sympatric speciation is considered to be rare in nature and a topic of debate [6–11].

Islands provide a unique opportunity to understand the evolutionary processes behind diversification and speciation [12–14]. For example, the patterns of speciation in birds were largely elucidated using studies of island birds [14–16]. Even though islands can harbor sister species, there is little evidence for sympatric speciation producing such species assemblages [4, 17] especially in small islands. Madeiran storm-petrel [6, 10] and Atlantic finches [11] are few such examples. Large islands (e.g. Madagascar) often have a greater diversity of terrain and ecological niches that may allow more intra-island diversification ([18–20] but see [21]). This diversity provides enough barriers for isolation, which results in intra-island allopatric speciation. Small islands, however, usually do not carry such diversity in niches. Therefore when related species are found in small islands, they tend to be results of ‘multiple colonizations’ [17, 22, 23].

**Fig 1:**
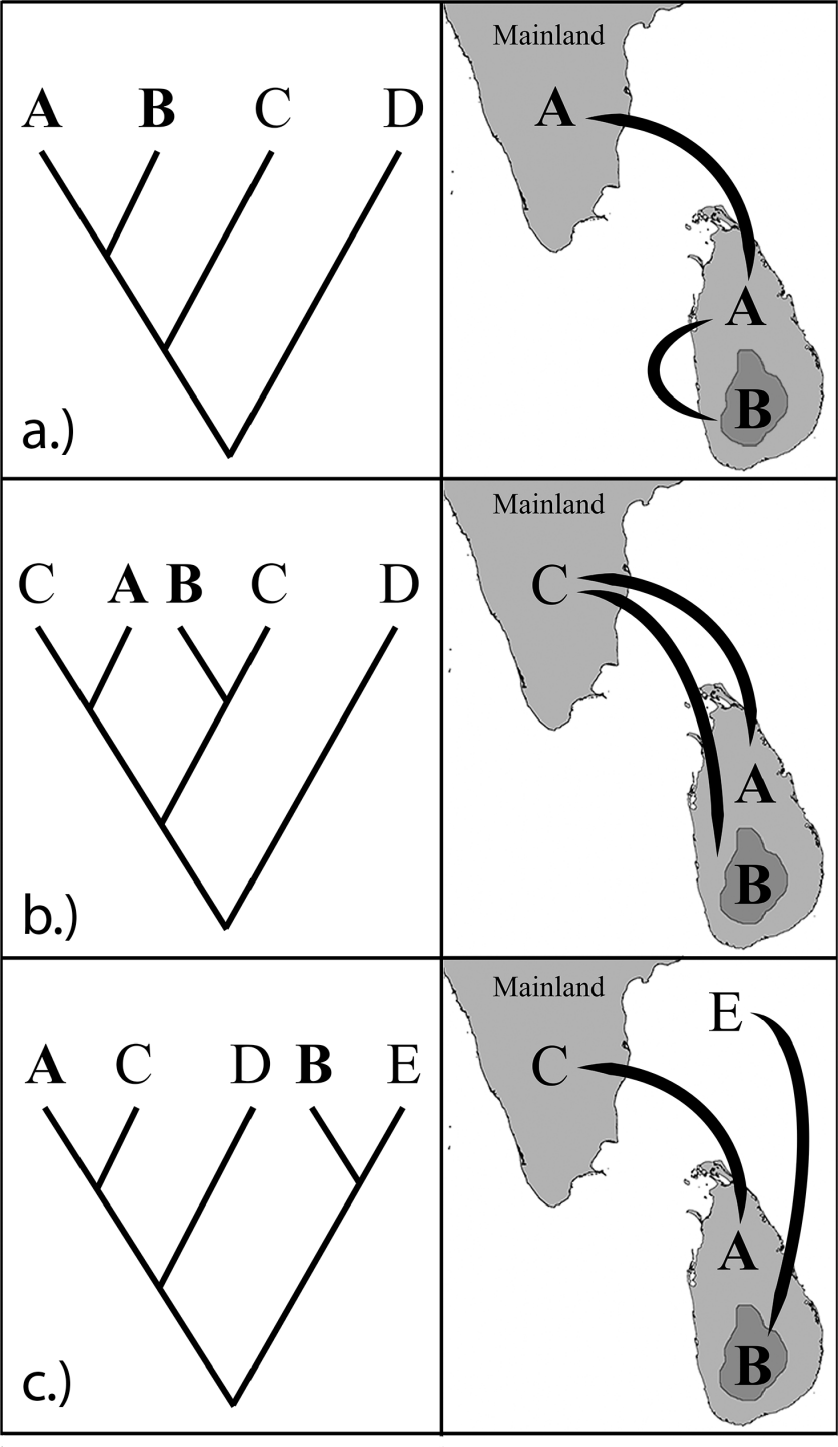
Three explanations for the co-occurrence of closely related taxa on an island. a.) Sympatric speciation/ intra-island diversification. The ancestor (A) from the mainland colonized the island. Later it got isolated and diverged into another species (B), resulting in two sister taxa (A and B). b.) Double colonization from same ancestral population (AB’), resulting in paraphyletic taxa (A and B). c.) Independent colonization from different ancestral populations (A and B), resulting in polyphyletic taxa.

Multiple colonization is a result of an island getting colonized more than once (twice—double colonization; more than twice—multiple colonization) by a foreign population. If there is sufficient time passed between such colonizations, they could diversify into separate species and co-exist in the island [15, 24, 25]. Such multiple colonizations can be seen across narrow water gaps in oceanic and continental islands [18, 26]. When these colonizations take place from the same ancestral population it results in species from a paraphyletic group co-occurring in islands (Fig 1). Ripley in 1949 suggested several such examples of probable double colonizations of birds into Sri Lanka from India including two species of barbets (the endemic *Megalaima rubricapilla* and the widespread *M*. *haemacephala*) two species of hill-mynahs (the endemic *Gracula ptilogneys* and the widespread *G*. *religiosa*) and two species of white-eyes (the endemic *Zosterops ceylonensis* and the widespread *Z*. *palpebrosus*) [25]. However, when colonization takes place from mainland populations with different ancestors (non-related populations) through independent colonization events, it could result in non-sister likely polyphyletic groups co-occurring in islands [17, 27–29] (see Fig 1).

Sri Lanka is a continental island situated on the same shallow continental shelf with India [30]. During Pleistocene glaciations, genetic mixing between Sri Lanka and the mainland (India) was possible through faunal exchange over the Palk Strait land bridge that emerged as a result of reduced sea level [31]. Sri Lankan biota is considered closely related to that of Southern India [32]. Together with the Western Ghats (of India) it is also considered a single biodiversity hotspot suggesting a single biogeographic community of species [32]. However, historical [33] as well as modern authors [34] recognize Sri Lankan biota as a distinct unit. Modern analyses using relatively less vagile groups such as freshwater crabs, freshwater fish, tree frogs and reptiles [34] had shown that Sri Lanka has its own endemism.

White-eyes are canopy-dwelling, small passerine birds belonging to the family Zosteropidae [35]. As the name implies, many have conspicuous white-colour eye rings, olive-green upper parts, yellow throats, and yellow or greyish-white bellies [35]. The family comprises of ~100 species belonging to 14 genera, of which the most speciose genus, *Zosterops* consists of typical white-eyes with ~75 species [35]. Fifty out of the 75 of these *Zosterops* white-eyes are island endemics indicating a high level of island endemism [35]. Many inhabit tropical islands in the Indian Ocean, western Pacific Ocean, and the Gulf of Guinea. They show high dispersal potential hence have the ability to colonize islands [28, 36, 37]. Sympatric occurrence of multiple species of white-eyes on islands is largely attributed to multiple colonizations as in the case of Mascarene Islands, Lord Howe Island and Norfolk Island white-eyes [17, 27–29]. It is suggested that dispersal of white-eyes took place from Asia to Africa [38], and from Asia or Australia into the central pacific [28, 36].

The Zosteropidae radiation took place ~2 Mya [36] and within this short period of time a rapid diversification of the *Zosterops* has taken place showing the highest rate of diversification among all vertebrates [36, 39]. As a result the white-eyes have been referred to as ‘great speciators’ in birds [28, 36, 40, 41]. They have been extensively used as models in bird speciation and evolutionary studies especially on islands [21, 37, 42–44].

**Fig 2:**
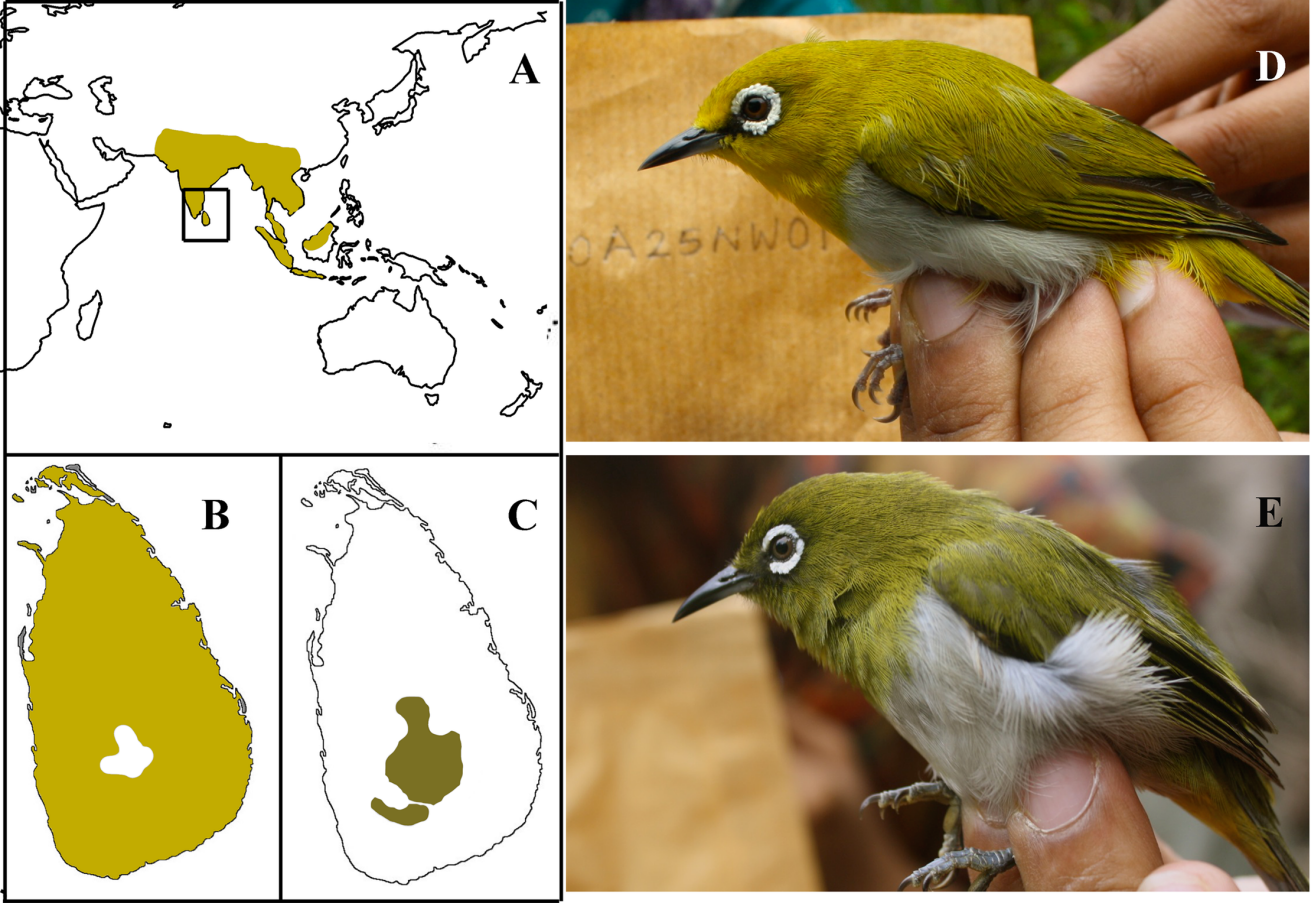
The two white-eye species in Sri Lanka. (A) Distribution of *Z*. *palpebrosus* and *Z*. *ceylonensis*. *Z*. *palpebrosus* has a widespread distribution throughout the oriental region and in Sri Lanka. The endemic *Z*. *ceylonensis* is confined to the hills of Sri Lanka. (B) *Z*. *palpebrosus* (Oriental white-eye) and (C) *Z*. *ceylonensis* (Ceylon white-eye) distribution in Sri Lanka. (D) *Z*. *palpebrosus* and (E) *Z*. *ceylonensis*.

The two species found in Sri Lanka are the endemic *Z*. *ceylonensis* (Ceylon White-eye or Hill White-eye) and its widespread congener *Z*. *palpebrosus* (Oriental White-eye) [45–47]. *Z*. *p*. *egregious* in Sri Lanka is a subspecies that has a widespread distribution throughout the oriental region including lowlands of India and Lakshadweep islands [35, 47, 48] (Fig 2). *Z*. *ceylonensis* is confined to the hills of Sri Lanka mainly above 1000m, common in high elevation evergreen forests, adjacent tea plantations and home gardens (Fig 2). Mees [48] stated that phenotypically *Z*. *ceylonensis* is much closer to *Z*. *p*. *nilgiriensis* (subspecies confined to the Nilgiri and Palani hills of the southern Western Ghats) than to other *Z*. *palpebrosus* and considered *Z*. *p*. *nilgiriensis* a link between *Z*. *palpebrosus* and *Z*. *ceylonensis* [48]. As previously mentioned, Ripley [25] suggested that the *Zosterops* white-eye species pair in Sri Lanka owe their origin to a double colonization from the same ancestral population in India.

The three possible scenarios that could explain the origin of the two white-eye species in Sri Lanka are through intra-island speciation (sympatric), double colonization from related mainland population in different time periods and independent colonization from different ancestral populations (Fig 1). In order to investigate the probable means of speciation, we performed phylogenetic analyses using gene sequence data to investigate their probable origin and colonization histories. Aim of our study was to answer three specific questions: 1.) are the two commonly known forms of white-eyes in Sri Lanka, *Z*. *ceylonensis* and *Z*. *palpebrosus* phenotypically and phylogenetically distinct? 2.) if so, are they phylogenetically sister to each other? and 3.) did *Z*. *ceylonensis* and *Z*. *palpebrosus* originate through sympatric speciation, double colonization from the same ancestral population or independent colonization from different (unrelated) populations?

## Methods

### Field sampling

The Department of Wildlife Conservation of Sri Lanka (Permit No: WL/3/2/19/13) reviewed the ethical, conservation and legal standing of this study and provided the permit to carry out the research. The Forest Department of Sri Lanka (Permit No: R&E/RES/NFSRC/14) allowed access to certain protected areas. The Forest Departments of Kerala (Permit No: Wl10-1647/2011) provided permits to carry out this study in Western Ghats of India.

**Fig 3:**
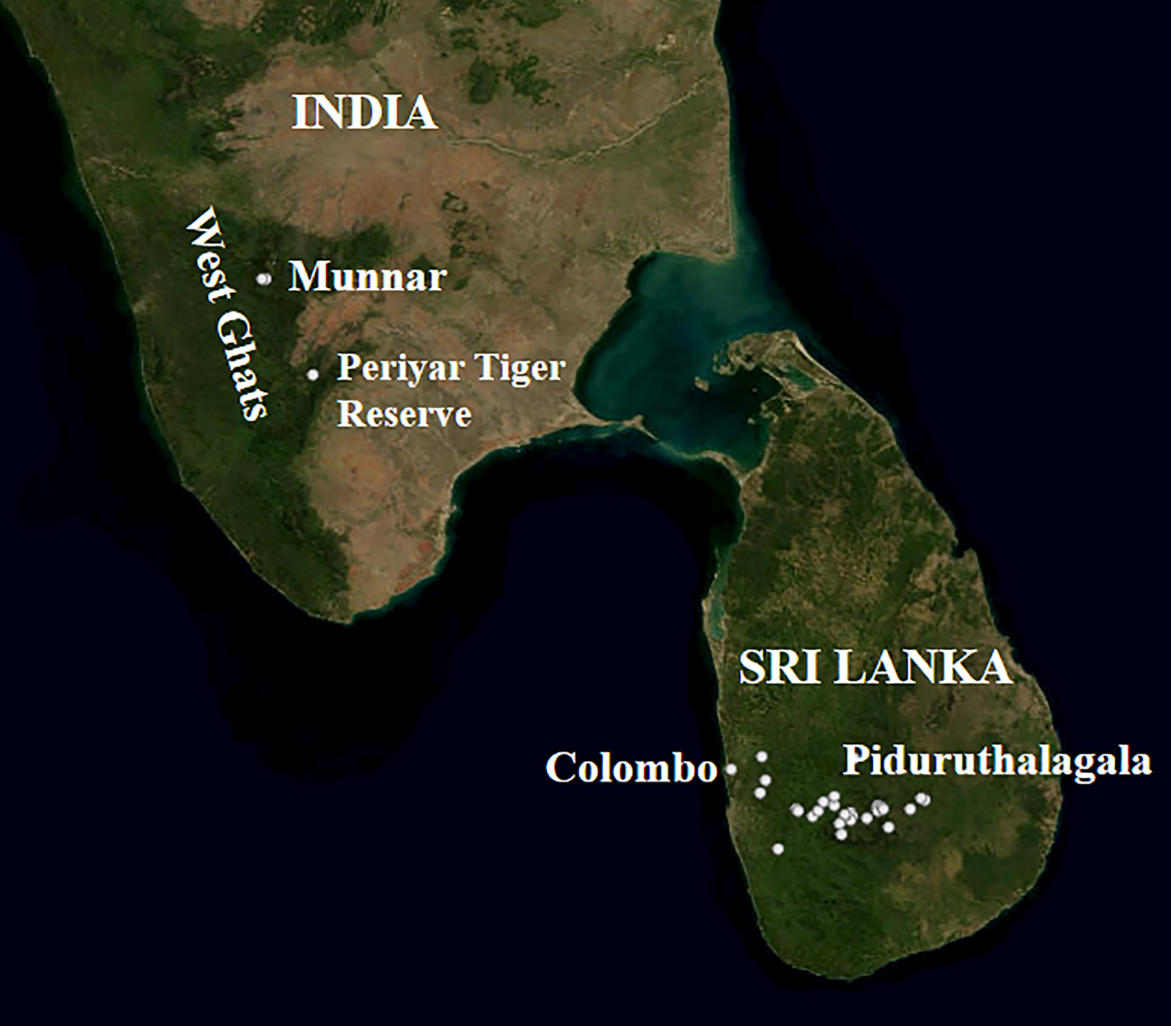
Sampling locations in Sri Lanka and in India. In Sri Lanka sampling spanned along an elevational gradient, from the sea level (0m) to the peak of the highest mountain, Piduruthalagala (2360m). In India sampling was carried out in Munnar (1800m-2500m) and in Periyar (800 -1000m).

Adult white-eyes were sampled using 12m mist nets of 16mm mesh size [49], with the help of call playbacks (as in [50]). In Sri Lanka, a total of 70 birds were captured in a ~200km transect along an elevational gradient spanning from the sea level (0m) at Colombo (6° 55' 37.48"N and 79° 51' 40.47"E) to 2367m at mount Piduruthalagala (7° 00' 03"N and 80° 46' 26"E) the highest peak in the island. In India, sampling was carried out in two locations in the Anaimalai Hills of the Western Ghats mountain range (Fig 3). A total of 10 birds were captured from Munnar (10° 05' 21"N and 77° 03' 35"E; 1800m-2500m) and from Periyar (9° 28' N and77° 10' E; 800 m-1000m). From each captured bird ~10μl of blood was collected from the brachial vein of the wing [51] and stored in Queen’s Lysis Buffer [52] and birds were released back to their original habitat.

### Phenotypic measurements

Fifteen morphological characters were measured using a dial caliper (±0.01mm) from each bird as in previous studies [50, 53] (S1 Fig). The measurements that could vary with the measurer were taken three times to reduce measurement error. NW took the measurements from all birds in this study.

### Genetic data

Genomic DNA was extracted using standard Phenol-Chloroform method (as in [50, 54]). The entire second and third subunits of mitochondrial nicotinamide adenine dinucleotide dehydrogenase (ND2 and ND3) and fifth intron of the nuclear gene transcription growth factor (TGFβ2) were amplified by polymerase chain reaction (PCR) and sequenced using Sanger sequencing method. The thermo cycling profile was as below: denaturation at 95°C for 5 min, followed by 35 cycles of denaturation at 94°C for 35s, annealing at 55°C for 35s, extension at 72°C for 45s, and a final extension step of 72°C for 15min. ND2 was PCR amplified using primers L5216 and H6313 [55], L5758 and H5766 [56], with the last two as internal sequencing primers. ND3 was amplified with the primers L10755 and H11151 [57] and TGFβ2 with TGF5-TGF6 [58]. Molecular work was done at the Molecular Ecology and Evolution laboratory at the Department of Zoology, University of Colombo and at the National Centre for Biological Sciences (NCBS), Bangalore. The Sequencing Facility at NCBS carried out Sanger sequencing for all the samples.

### Data analysis

#### Phenotypic analysis

Discriminant Function Analysis (DFA) was performed on the phenotypic data to determine whether there is a distinct phenotypic clusters, corresponding the existing species of the white-eyes of Sri Lanka, in the dataset. To avoid collinear variables, significant principal components (PCs) that resulted from a principal component analysis were selected using eigenvalues and scree plots [59]. Two canonical plots were derived (JMP ver. 8, SAS Inst., Cary, NC), one with the reduced set of variables (which had the highest contribution to the selected PCs) and the second using the selected PCs.

#### Phylogenetic analysis

We used Geneious version 7.1.6 [60] to examine the trace files for quality, to edit sequences, *de novo* assemble and multiple align sequences across taxa using ClustalW algorithm [61]. We examined appropriate models of evolution and the best way to partition gene regions using PartitionFinderver 1.1.0 [62]. The optimal partitioning scheme had 4 partitions: 1^st^ codon position ND2 and ND3, 2^nd^codon position ND2 and ND3, 3^rd^ codon position ND2 and ND3, and TGF. Phylogenetic trees were built through Maximum likelihood (ML) approach using RAxML ver. 8.1.22 [63] and Bayesian approach using MrBayes ver 3.2.5 [64].

For ML, we conducted tree searches rapid bootstrap of 1000 replicates and there after a thorough ML search of 10 runs using a separate GTR+G+I evolutionary model for each partition. Invariant sites were not included in the model. We conducted a Bayesian analysis by running the MCMC chain for 20,000,000 generations, sampling every 1000 steps, with 25% of the samples discarded as burnin. We assessed the convergence using the standard deviation of split frequencies below 0.01 and checking for stationarity using Tracer ver. 1.6 [65].

### Divergence dating

We used BEAST ver. 2.4.4 [66] to estimate divergence times within Zosteropidae. We assigned HKY model for each gene, with 4 categories estimate shape for gamma. We used a second calibration of Zosteropidae + *Zosteronis* (formerly *Stachyris*) from Philippines cited in Moyle et al [36]—5.01 Ma (4.46–5.57 Ma). We assumed a Yule speciation process for the tree model and a relaxed clock lognormal distribution for the molecular clock model, and linked clock and tree models. We set calibration as a normal distribution with mean 5.01 and sigma of 0.555 and ran MCMC chains for 20 million generations, sampling every 500^th^ generation and discarding the first 25% as burnin. We used Tracer ver. 1.6 [65] to examine parameters and to ensure stationarity.

## Results

### Phenotypic analysis

**Fig 4:**
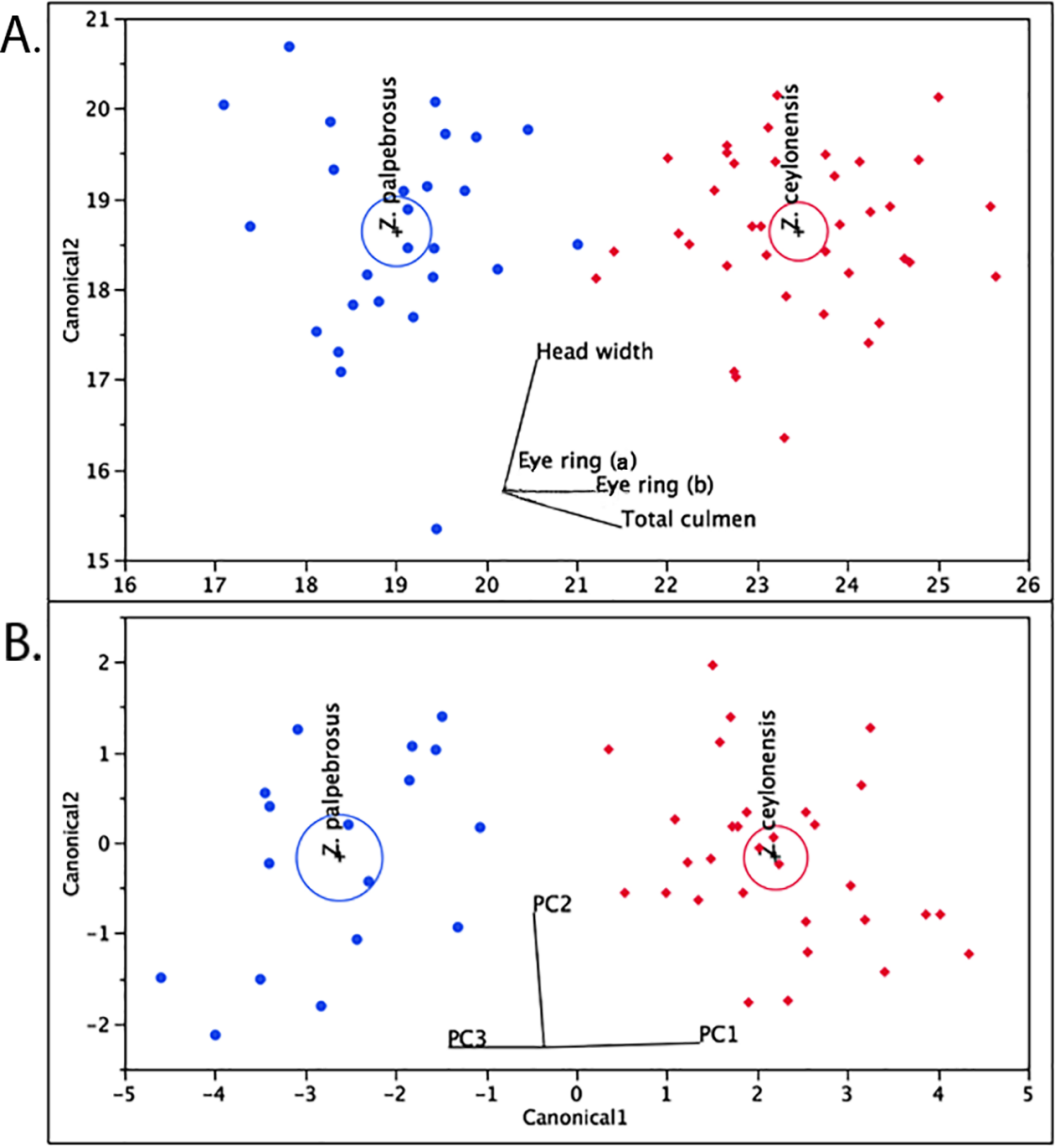
Canonical plots derived from the multivariate analysis. **(A)** Canonical plot of phenotypic variation of the *Z*. *ceylonensis* and *Z*. *palpebrosus* populations with the morphological features that significantly contributed to the three reduced variables (PC1-PC3). *Z*. *ceylonensis* and *Z*. *palpebrosus* are two phenotypically distinct clusters that differentiate along the horizontal axis. Eye ring opening and total culmen contributed significantly to differentiating the species along this axis. (**B)** Canonical plot of phenotypic variation of the *Z*. *ceylonensis* and *Z*. *palpebrosus* populations with PC1, PC2 and PC3. *Z*. *ceylonensis* and *Z*. *palpebrosus* are two phenotypically distinct clusters for which PC1 contributed significantly.

Based on the eigenvalues (S2 Table) and scree plot (S2 Fig) first three PCs were selected for the DFA. The canonical plots separated *Z*. *ceylonensis* and *Z*. *paplebrosus* into two phenotypically distinct clusters with 87% accuracy (Fig 4). Opening of the eye ring and total culmen significantly contributed to the phenotypic distinctness of the two species (Fig 4).

### Phylogenetic analysis

**Fig 5:**
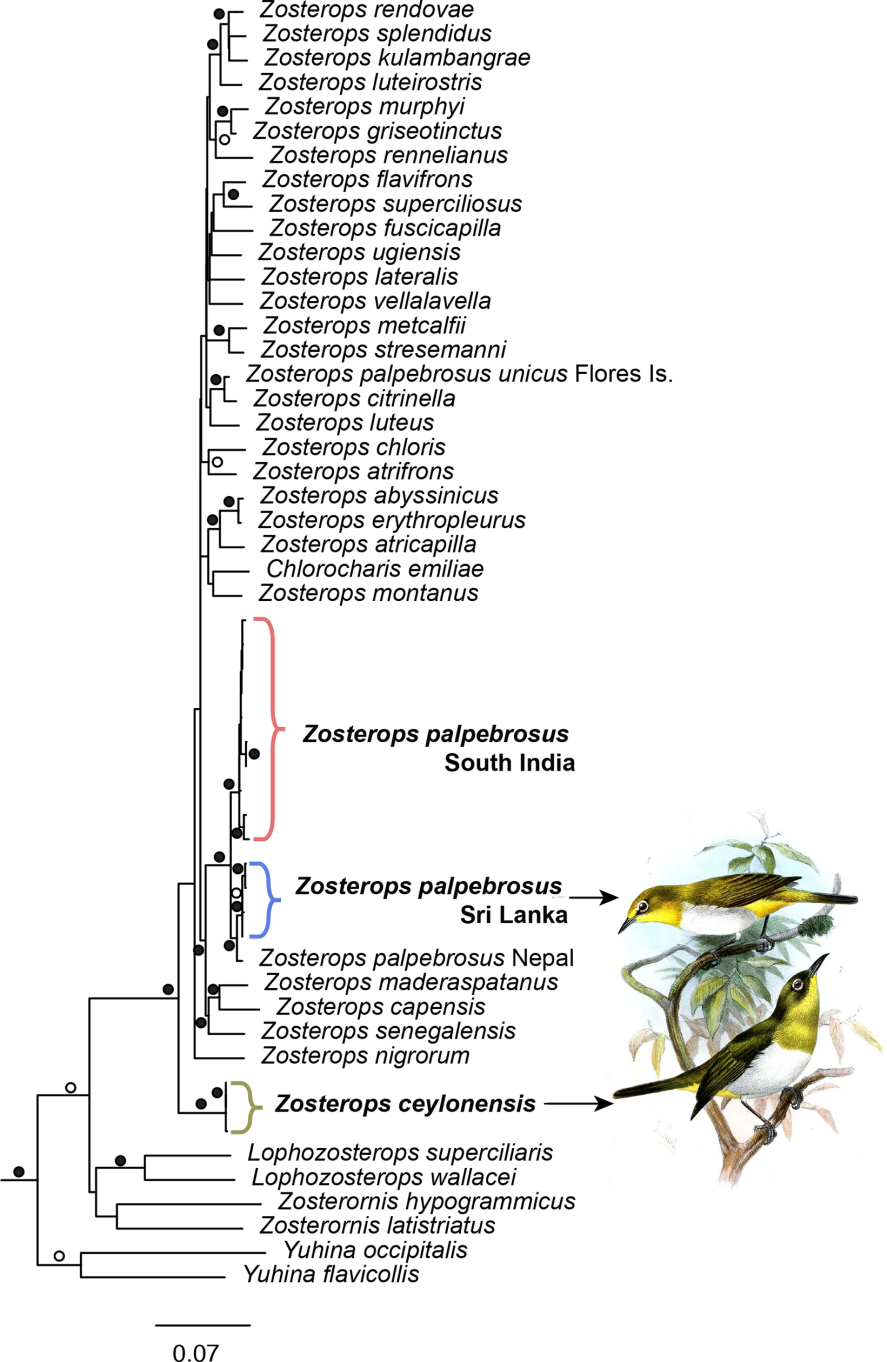
Phylogenetic affinities of white-eyes. Phylogenetic relationships of *Zosterops* white-eyes using maximum likelihood (ML) analyses of the concatenated, partitioned dataset of 3 genes (ND2, ND3, TGF). The topology of ML and Bayesian analyses were highly similar (outgroups not shown). Symbols at nodes indicate ML bootstrap support (open circles show >70%, solid circles show >90%); all nodes with circles had Bayesian posterior probabilities values of 0.95 or greater. Illustrations of white-eyes are by J. Smit [67] (public domain).

The concatenated and partitioned ML and Bayesian trees showed similar topologies. In both analyses, there are several short internodes and polytomies, however, the clades of significance to Sri Lankan birds were well supported (Fig 5). *Z*. *ceylonensis* did not cluster with sympatric *Z*. *palpebrosus* but is resolved as the basal lineage to all its congeners in the clade of *Zosterops*. This relationship received high ML bootstrap support (100%) and Bayesian posterior probability (1.0). *Zosterops palpebrosus* is not monophyletic, *Z*. *p*. *unicus* (Flores Island) groups with Australasian species while the southern Asian sub species are in a separate clade that is sister to Western Indian Ocean (African) species with strong support (90% ML bootstrap/ 1.0 Bayesian PP). The Sri Lanka population of *Z*. *palpebrosus* (currently in the widespread subspecies (*egregius*) is sister to the Western Ghats population (*Z*. *p*. *nilgiriensis*) with strong support (100/ 1.0). Individual gene trees of ND2 and ND3 show similar patterns to the concatenated analyses in both ML and Bayesian approaches, however, the topology using TGF5 is less resolved, perhaps due to insufficient informative sites.

### Divergence dating

**Fig 6:**
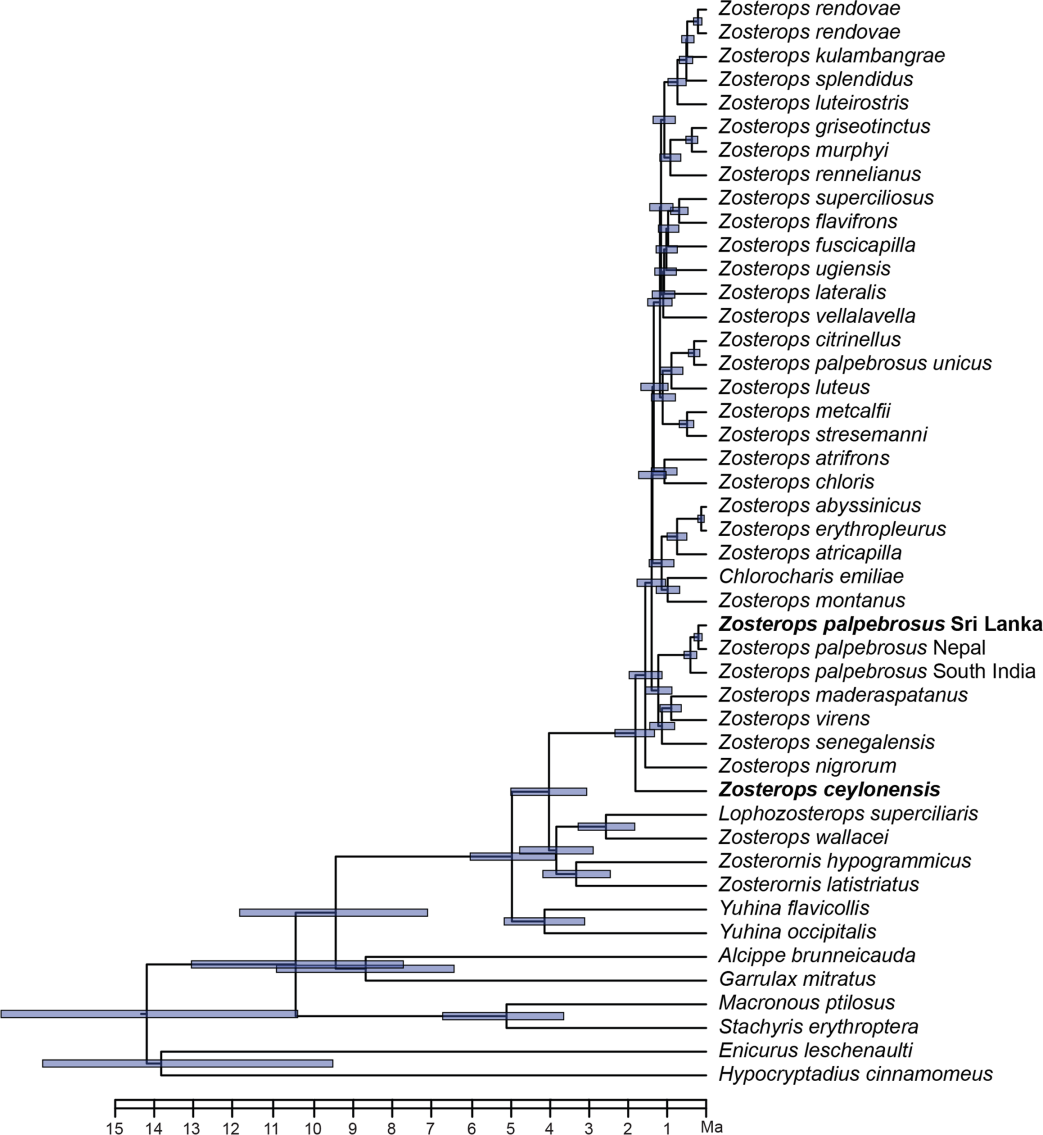
Divergence times of *Zosterops*. The blue colour bars indicate 95% HPD (Highest Posterior Density) intervals. Divergence time estimates show that *Z*. *ceylonensis* diverged around 1.79 MYA and *Z*. *palpebrosus* in Sri Lanka diverged around 0.19 MYA.

Results from the BEAST dating analyses (Fig 6) shows that *Z*. *ceylonensis*is much older divergence that split from its congeners around 1.79MYA (95% HPD: 1.31–2.32). *Zosterops palpebrosus* in Sri Lanka, on the other hand, is a more recent split that diverged 0.19MYA (95% HPD: 0.10–0.31) from its sister population.

## Discussion

### Phenotypic distinctness of *Z*. *ceylonensis* and *Z*. *palpebrosus* in Sri Lanka

Differences in phenotype (mainly plumage and vocalization), which had been the basis for separating the two species, is known for *Z*. *ceylonensis* and *Z*. *palpebrosus* in Sri Lanka [35, 45–47]. With an unbiased character based approach, here we showed that the two species are distinct phenotypic clusters (Fig 4). Mees [48] suggested that *Z*. *ceylonensis* is morphologically closer to *Z*. *p*. *nilgiriensis* than any other *Z*. *palpebrosus* [48], our study did not investigate morphological similarities of *Z*. *ceylonensis* and *Z*. *p*. *nilgiriensis*, however our phylogenetic analysis showed a separate origin for these two lineages.

### Are *Z*. *ceylonensis* and *Z*. *palpebrosus* in Sri Lanka phylogenetically distinct?

All individuals identified as *Z*. *ceylonensis* grouped together as a monophyletic group. Similarly, all individuals of *Z*. *palpebrosus* from Sri Lanka form a distinct group. Therefore *Z*. *ceylonensis* and *Z*. *palpebrosus* formed two distinct clades (Fig 5). There is concordance between morphometric and phylogenetic divergence across these two species (Figs 4–6), hence we conclude that they are phenotypically and phylogenetically distinct lineages and true species for Sri Lanka.

### Are *Zosterops* white-eyes in Sri Lanka sister to each other?

Our analysis shows that these two lineages of Sri Lankan white-eyes are not each other’s closest relatives. Similar to other studies of white-eyes [28, 36], the genus *Zosterops* likely diversified very rapidly as implied by the extremely short internodes throughout much of the tree (this study and in [28, 36]). Nevertheless, we can draw conclusions based on strong nodal support values regarding the placement of the Sri Lankan lineages. *Z*. *ceylonensis* is not closely related to the *Z*. *palpebrosus*, which is the most geographically proximate species found throughout southern Asia and in Sri Lanka, but rather is sister to the entire *Zosterops* clade (Figs 5 and 6). There is strong support for this relationship in our phylogeny but given that many other named species of *Zosterops* are yet to be sampled genetically, additional data will shed more light into this relationship.

Furthermore there is strong support for the placement of Sri Lankan *Z*. *p*. *egregious* as sister to the Western Ghats *Z*. *p*. *nilgiriensis* (Fig 5). However *Z*. *palpebrosus* appears to be polyphyletic with at least one subspecies (*Z*. *p*. *unicus*) not grouping with the remaining populations from Asia. We only have a limited number of populations included from southern Asia to the analysis, but within this sampling, the southern populations from Sri Lanka and Western Ghats are more closely related than populations within the rest of Indian subcontinent. *Z*. *palpebrosus* is sister to a clade from western Indian Ocean islands and Africa with strong support, but the directionality of colonization is unclear given the short internodes of other congeners (Figs 5 and 6) [28].

### Did *Z*. *ceylonensis* and *Z*. *palpebrosus* in Sri Lanka originate through sympatric speciation, double colonization from same population or independent colonization from different populations?

Ripley (1949) suggested the double ‘invasion’ (colonization) hypothesis to explain the origins of the white-eye species pair in Sri Lanka and assumed *Z*. *ceylonensis* as the species that may have arrived first to the island [25]. Our results, especially the dated phylogeny confirms the early arrival of *Z*. *ceylonensis* (Fig 6). Moreover our phylogenetic analysis indicates that the Sri Lankan *Z*. *palpebrosus* show strong affinities to the Indian *Z*. *palpebrosus*. However *Z*. *ceylonensis* does not show affinities to any of the extant south Asian clades. Therefore the Sri Lankan white-eyes must have colonized from different ancestral source in different time windows.

## Conclusions

Here we showed that *Z*. *ceylonensis* and *Z*. *palpebrosus* in Sri Lanka are phenotypically and genetically distinct entities, and that they are not sister to each other. *Z*. *palpebrosus* is sister to the Western Indian Ocean *Zosterops* clade and within, *Z*. *p*. *egregius* in Sri Lanka is sister to *Z*. *p*. *nilgiriensis* of Western Ghats. Our results suggest that the two *Zosterops* species originated in the island through independent colonizations from different ancestral lineages and not through island speciation or double colonization from the same continental ancestral population. We also confirm that *Z*. *ceylonensis* is an ancient lineage which originated first and *Z*. *palpebrosus* later. While the origin of *Z*. *ceylonensis* is still unclear, our results imply that *Z*. *ceylonensis* could be the ancestor to all *Zosterops* white-eyes. However, due to the fact that many of the *Zosterops* have not been sampled, identity of the ancestral *Zosterops* cannot be confirmed. This study provides vital information on the patterns of speciation and the generation of endemism in the island of Sri Lanka. It stresses that Sri Lanka fauna may not entirely be a subset of the Indian faunal assemblage, even with groups that show high dispersal ability such as birds. The patterns of colonization can get complicated in continental islands with a history of complex geological affinities with neighboring landmasses.

## Supporting information

S1 Fig Morphometric measurements used for the morphometric analysis. 1. weight 2. head length 3. head width 4. Total culmen 5. Exposed culmen 6. bill height 7. bill width 8. thickness of the eye-ring; eye ring (a) 9. opening of the ring; eye ring (b) 10. diameter of the eye-ring; eye ring (c) 11. eye ring width 12. flattened wing length 13. tarsus (right) length 14. first claw length 15. tail length.
(TIF)

S2 Fig Scree plot. This plots the eigen values associated with each PC. At PC4 the slope of the curve levels off, hence only PC1, PC2 and PC3 were used for the analysis.
(TIF)

S1 Table Sample ID or band number, tissue source and collection locality for each species used in the phylogenetic study with GenBank accession numbers for each gene sequence. Footnote.
FMNH, The Field Museum of Natural History; KUNHM, University of Kansas Natural History Museum; LSUMNS, Louisiana State University Museum of Natural Science; USNM, National Museum of Natural History; UWBM, University of Washington Burke Museum; AMNH, American Museum of Natural History; CMNH, Cleveland Museum of Natural History.
(DOCX)

S2 Table Eigen values for each variable in each principal component (PC) resulted from the principal component analysis. (DOCX)
