# Supplementary figures and images for: Non-sister Sri Lankan white-eyes (genus Zosterops) are a result of independent colonizations
Source: PLoS One. 2017 Aug 9;12(8):e0181441. doi: 10.1371/journal.pone.0181441 (PMC5549887; doi:10.1371/journal.pone.0181441)

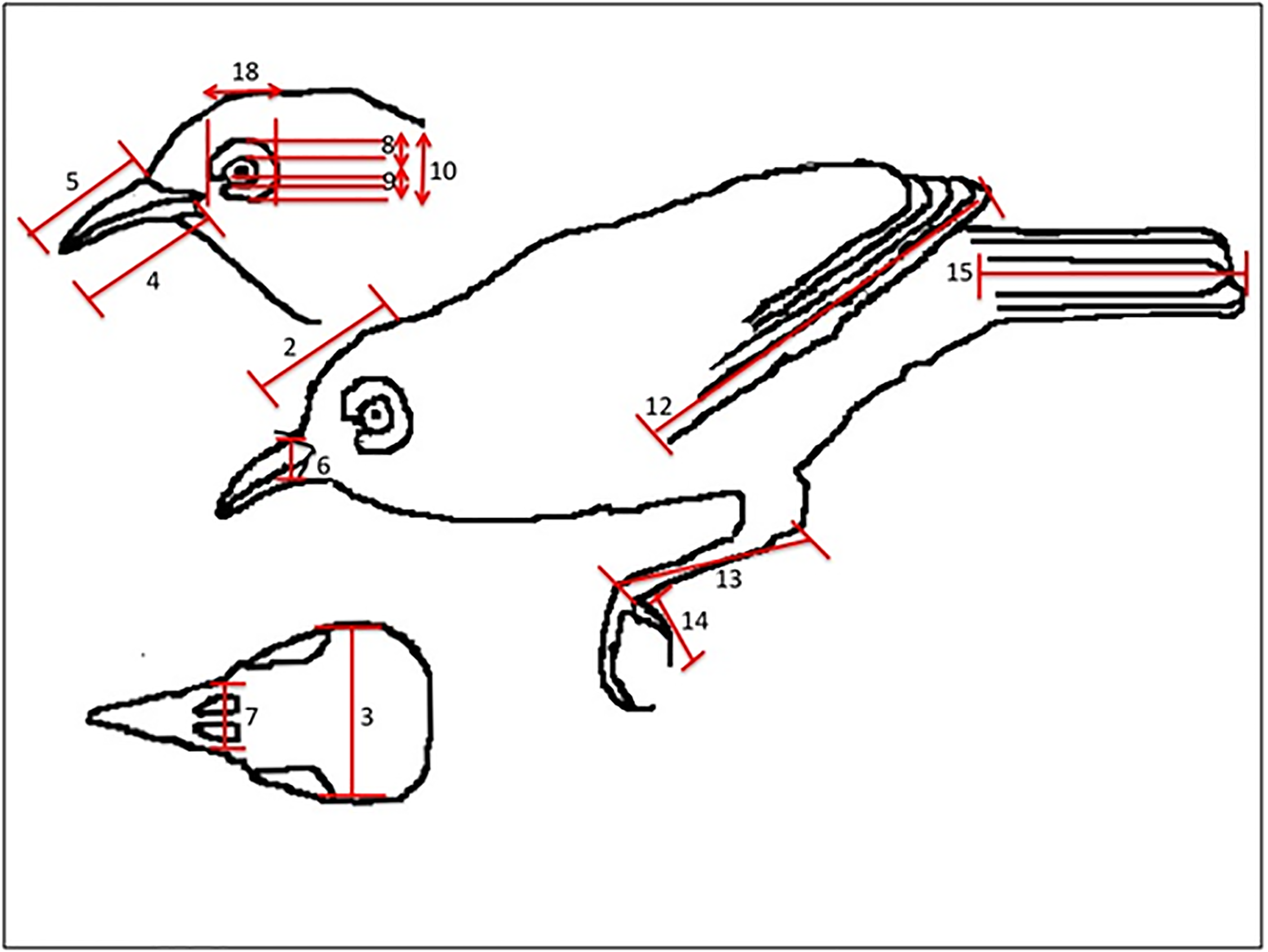

Supplement: S1 Fig — 1. weight 2. head length 3. head width 4. Total culmen 5. Exposed culmen 6. bill height 7. bill width 8. thickness of the eye-ring; eye ring (a) 9. opening of the ring; eye ring (b) 10. diameter of the eye-ring; eye ring (c) 11. eye ring width 12. flattened wing length 13. tarsus (right) length 14. first claw length 15. tail length. (TIF) [file pone.0181441.s001.tif]

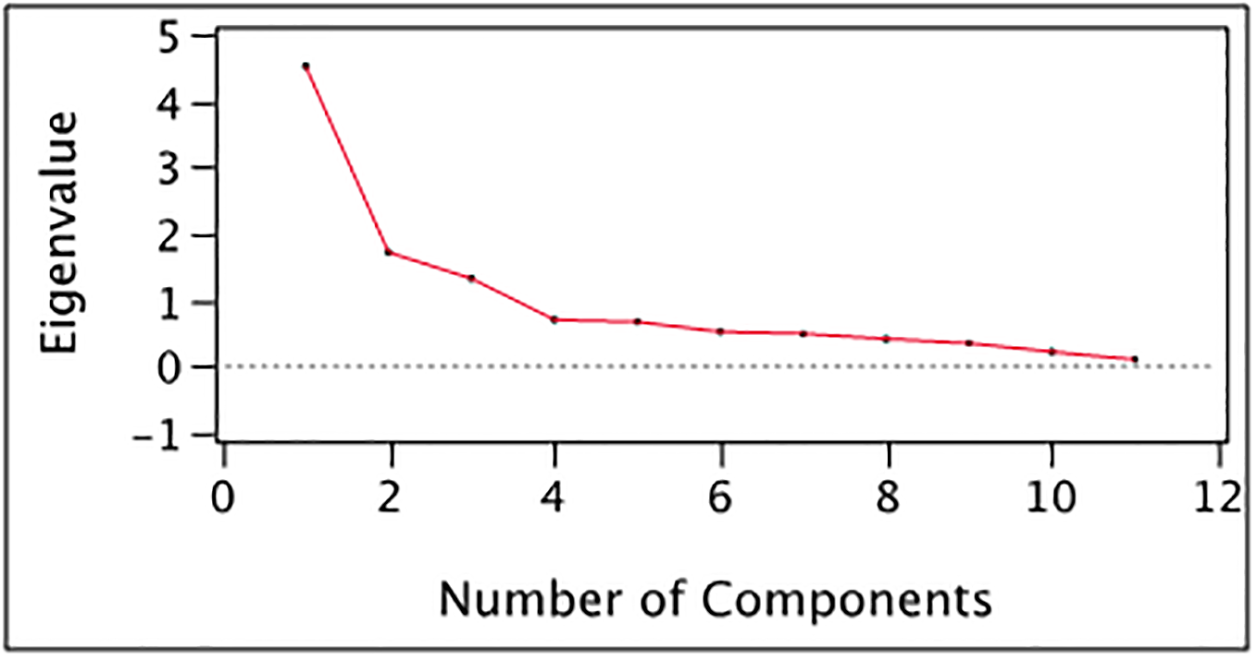

Supplement: S2 Fig — This plots the eigen values associated with each PC. At PC4 the slope of the curve levels off, hence only PC1, PC2 and PC3 were used for the analysis. (TIF) [file pone.0181441.s002.tif]
