# Supplementary material for: Non-sister Sri Lankan white-eyes (genus Zosterops) are a result of independent colonizations
Source: PLoS One. 2017 Aug 9;12(8):e0181441. doi: 10.1371/journal.pone.0181441 (PMC5549887; doi:10.1371/journal.pone.0181441)
Supplement: S1 Table — Footnote. FMNH, The Field Museum of Natural History; KUNHM, University of Kansas Natural History Museum; LSUMNS, Louisiana State University Museum of Natural Science; USNM, National Museum of Natural History; UWBM, University of Washington Burke Museum; AMNH, American Museum of Natural History; CMNH, Cleveland Museum of Natural History. (DOCX) [file pone.0181441.s003.docx]

**S1 Table:**

**Sample ID or band number, tissue source and collection locality for each species used in the phylogenetic study with GenBank accession numbers for each gene sequence**

| **Species** | **Locality** | **Source** | **Sample ID/Band No./Voucher No.** | **ND2** | **ND3** | **TGF** |
| --- | --- | --- | --- | --- | --- | --- |
| *Zosterops palpebrosus* | Periyar, Thekkady | This study | Z51603 | KY765107 | KY765147 | KY765127 |
| *Zosterops palpebrosus* | Periyar, Vallakadavu | This study | Z51507 | KY765109 | KY765148 | KY765129 |
| *Zosterops palpebrosus* | Munnar, Rajamalai Shola | This study | Z51241 | KY765104 | KY765144 | KY765124 |
| *Zosterops palpebrosus* | Munnar, Rajamalai Shola | This study | Z51123 | KY765101 | KY765141 | KY765121 |
| *Zosterops palpebrosus* | Periyar, Vallakadavu | This study | Z51501 | KY765108 | - | KY765128 |
| *Zosterops palpebrosus* | Periyar, Vallakadavu | This study | Z51509 | KY765110 | KY765149 | KY765130 |
| *Zosterops palpebrosus* | Munnar, Rajamalai Shola | This study | Z51129 | KY765102 | KY765142 | KY765122 |
| *Zosterops palpebrosus* | Munnar, Signal Station | This study | Z51227 | KY765103 | KY765143 | KY765123 |
| *Zosterops palpebrosus* | Munnar, Signal Station | This study | Z51229 | - | KY765146 | KY765126 |
| *Zosterops palpebrosus* | Munnar, Signal Station | This study | Z51228 | KY765105 | KY765145 | KY765125 |
|  | Kotadeniyawa, Puwakwaththa | This study | MG17SF05 | KY765100 | - | KY765120 |
| *Zosterops palpebrosus* | Galle, Walahanduwa | This study | MD18SS04 | KY765098 | - |  |
| *Zosterops palpebrosus* | Pilikuththuwa, Gampaha | This study | ME28SF02 | KY765139 | - | KY765118 |
| *Zosterops palpebrosus* | Nuwaraeliya, Sandathenna | This study | NE09NW01 | KY765095 | KY765136 | KY765115 |
| *Zosterops palpebrosus* | Nuwaraeliya, Sandathenna | This study | NE09NW02 | KY765096 | KY765137 | KY765116 |
| *Zosterops palpebrosus* | Gampaha, Ja-ela | This study | MK22SF01 | KY765099 | KY765140 | KY765118 |
| *Zosterops palpebrosus* | Nuwaraeliya, Maskeliya | This study | NF02NW03 | KY765097 | KY765138 | KY765117 |
| *Zosterops palpebrosus* | Nuwaraeliya, Sandathenna | This study | NE11NW01 | KY765094 | KY765135 | KY765114 |
| *Zosterops ceylonensis* | Nuwaraeliya, Pattipola | This study | NE10NW04 | KY765093 | KY765134 | KY765113 |
| *Zosterops ceylonensis* | Nuwaraeliya, Pattipola | This study | NE10NW03 | KY765092 | KY765133 | KY765112 |
| *Zosterops ceylonensis* | Nuwaraeliya, Piduruthalagala | This study | MH14SF03 | KY765090 | KY765131 | KY765111 |
| *Zosterops ceylonensis* | Nuwaraeliya, Piduruthalagala | This study | MH14SF04 | KY765091 | KY765132 | - |
| *Chlorocharis emiliae* | Borneo | Moyle et al. 2009 | LSUMNS B51361 | FJ460796 | FJ460864 | FJ460933 |
| *Zosterops montanus* | Sulawesi, Indonesia | Moyle et al. 2009 | AMNH DOT12552 | FJ460810 | FJ460878 | FJ460947 |
| *Zosterops abyssinicus* | Captive | Moyle et al. 2009 | LSUMNS B20825 | FJ460799 | FJ460867 | FJ460936 |
| *Zosterops erythropleura* | Captive | Moyle et al. 2009 | LSUMNS B20626 | FJ460800 | FJ460868 | FJ460937 |
| *Zosterops atricapilla* | Borneo | Moyle et al. 2009 | LSUMNS B36444 | FJ460802 | FJ460870 | FJ460939 |
| *Zosterops pallidus* | South Africa | Linck et al. 2015 | MGD26 | KT310495 |  | KT310680 |
| *Zosterops virens* | South Africa | Moyle et al. 2009 | FMNH 390165 | FJ460811 | FJ460879 | FJ460948 |
| *Zosterops maderaspatanus* | Madagascar | Moyle et al. 2009 | FMNH 345980 | FJ460813 | FJ460881 | FJ460950 |
| *Zosterops senegalensis* | Ghana | Moyle et al. 2009 | LSUMNS B39250 | FJ460803 | FJ460871 | FJ460940 |
| *Lophozosterops goodfellowi* | Mindanao, Philippines | Moyle et al. 2009 | FMNH 357641 | FJ460792 | FJ460860 | FJ460929 |
| *Oculocincta squamifrons* | Borneo | Moyle et al. 2009 | LSUMNS B51197 | FJ460795 | FJ460863 | FJ460932 |
| *Zosterops wallacei* | Sumba Is., Indonesia | Moyle et al. 2009 | WAM 22903 | FJ460816 | FJ460884 | FJ460953 |
| *Lophozosterops superciliaris* | Flores, Indonesia | Moyle et al. 2009 | WAM 223291 | FJ460794 | FJ460862 | FJ460931 |
| *Lophozosterops squamiceps* | Sulawesi, Indonesia | Moyle et al. 2009 | AMNH DOT12549 | FJ460793 | FJ460861 | FJ460930 |
| *Stachyris hypogrammica* | Palawan, Philippines | Moyle et al. 2009 | CMNH 37765 | FJ460788 | FJ460856 | FJ460925 |
| *Stachyris hypogrammica* | Palawan, Philippines | Moyle et al. 2009 | FMNH 455063 | JN826670 | JN826940 | JN826414 |
| *Hypocryptadius cinnamomeus* | Mindanao, Philippines | Moyle et al. 2009 | FMNH 357652 | FJ460769 | FJ460837 | FJ460905 |
| *Enicurus leschenaulti* | Borneo | Moyle et al. 2009 | LSUMNS B36442 | AY878291 | AY878266 | FJ460904 |
| *Garrulax mitratus* | Borneo | Moyle et al. 2009 | LSUMNS B36460 | FJ460776 | FJ460844 | FJ460912 |
| *Garrulax mitratus* | Borneo | Moyle et al. 2012 | KUNHM 17728 | JN826518 | JN826777 | JN826267 |
| *Macronous ptilosus* | Borneo | Moyle et al. 2009 | LSUMNS B36391 | FJ460774 | FJ460842 | FJ460910 |
| *Macronous ptilosus* | Borneo | Moyle et al. 2009 | KUNHM 12325 | JN826573 | JN826834 | JN826320 |
| *Stachyris erythroptera* | Borneo | Moyle et al. 2012 | KUNHM 12327 | JN826668 | JN826938 | JN826412 |
| *Napothera crassa* | Borneo | Moyle et al. 2009 | LSUMNS B36469 | FJ460773 | FJ460841 | FJ460909 |
| *Napothera crassa* | Borneo | Moyle et al. 2012 | KUNHM 17805 | JN826599 | JN826862 | JN826346 |
| *Illadopsis rufipennis* | Ghana | Moyle et al. 2012 | KUNHM 15583 | JN826557 | JN826816 | JN826303 |
| *Illadopsis rufipennis* | Ghana | Moyle et al. 2009 | LSUMNS B39437 | FJ460777 | FJ460845 | FJ460913 |
| *Illadopsis rufipennis* | Eq. Guinea | Moyle et al. 2012 | KUNHM 8577 | JN826558 | JN826817 | JN826304 |
| *Pellorneum capistratum* | Borneo | Moyle et al. 2009 | LSUMNS B36430 | FJ460772 | FJ460840 | FJ460908 |
| *Malacopteron magnirostre* | Borneo | Moyle et al. 2009 | LSUMNS B36421 | FJ460778 | FJ460846 | FJ460914 |
| *Malacopteron magnirostre* | Borneo | Moyle et al. 2012 | KUNHM 12358 | JN826585 | JN826847 | JN826333 |
| *Yuhina brunneiceps* | Taiwan | Moyle et al. 2009 | AMNH DOT5230 | FJ460782 | FJ460850 | FJ460918 |
| *Yuhina brunneiceps* | Taiwan | Moyle et al. 2012 | AMNH DOT5153 | JN826706 | JN826980 | JN826450 |
| *Yuhina nigrimentas* | Vietnam | Moyle et al. 2009 | AMNH DOT12297 | FJ460785 | FJ460853 | FJ460921 |
| *Yuhina nigrimenta* | China | Moyle et al. 2012 | KUNHM 11150 | JN826711 | JN826985 | JN826454 |
| *Yuhina castaniceps* | China | Moyle et al. 2009 | KUNHM 6676 | FJ460780 | FJ460848 | FJ460916 |
| *Yuhina castaniceps* | China | Moyle et al. 2012 | KUNHM 13784 | JN826708 | JN826982 | JN826451 |
| *Yuhina everetti* | Borneo | Moyle et al. 2009 | LSUMNS B36290 | FJ460779 | FJ460847 | FJ460915 |
| *Yuhina everetti* | Borneo | Moyle et al. 2012 | KUNHM 17756 | JN826709 | JN826983 | JN826452 |
| *Yuhina flavicollis* | Myanmar | Moyle et al. 2012 | KUNHM 15170 | JN826710 | JN826984 | JN826453 |
| *Yuhina occipitalis* | Nepal | Moyle et al. 2009 | AMNH DOT5588 | FJ460786 | FJ460854 | FJ460922 |
| *Yuhina occipitalis* | Myanmar | Moyle et al. 2012 | KUNHM 15177 | JN826713 | JN826987 | JN826456 |
| *Stachyris dennistouni* | Luzon, Philippines | Moyle et al. 2009 | CMNH 38201 | FJ460787 | FJ460855 | FJ460923 |
| *Stachyris dennistouni* | Philippines | Moyle et al. 2012 | KUNHM 20186 | JN826666 | JN826936 | JN826410 |
| *Stachyris dennistouni* | Philippines | Moyle et al. 2012 | KUNHM 20225 | JN826667 | JN826937 | JN826411 |
| *Stachyris capitalis* | Mindanao, Philippines | Moyle et al. 2009 | CMNH 37769 | FJ460791 | FJ460859 | FJ460928 |
| *Stachyris whiteheadi* | Philippines | Moyle et al. 2012 | FMNH 429219 | JN826688 | JN826961 | JN826432 |
| *Stachyris whiteheadi* | Philippines | Moyle et al. 2012 | KUNHM 18001 | JN826687 | JN826960 | JN826431 |
| *Stachyris latistriata* | Panay, Philippines | Moyle et al. 2009 | CMNH 34221 | FJ460790 | FJ460858 | FJ460927 |
| *Stachyris plateni* | Mindanao, Philippines | Moyle et al. 2009 | CMNH 37768 | DQ402187 | DQ402246 | FJ460924 |
| *Stachyris speciosa* | Negros, Philippines | Moyle et al. 2009 | CMNH 37091 | FJ460789 | FJ460857 | FJ460926 |
| *Zosterops nigrorum* | Luzon, Philippines | Moyle et al. 2009 | FMNH 432997 | FJ460808 | FJ460876 | FJ460945 |
| *Zosterops atrifrons* | Sulawesi, Indonesia | Moyle et al. 2009 | AMNH DOT12620 | FJ460809 | FJ460877 | FJ460946 |
| *Zosterops chloris* | Sulawesi, Indonesia | Moyle et al. 2009 | AMNH DOT12558 | FJ460798 | FJ460866 | FJ460935 |
| *Zosterops palpebrosus* | Flores Is., Indonesia | Moyle et al. 2009 | WAM 23218 | FJ460807 | FJ460875 | FJ460944 |
| *Zosterops citrinellus* | Roti Is., Indonesia | Moyle et al. 2009 | WAM 23542 | FJ460815 | FJ460883 | FJ460952 |
| *Woodfordia superciliosa* | Rennell, Solomon Is. | Moyle et al. 2009 | UWBM 58818 | FJ460797 | FJ460865 | FJ460934 |
| *Zosterops flavifrons* | Vanuatu | Moyle et al. 2009 | LSUMNS B45805 | FJ460805 | FJ460873 | FJ460942 |
| *Zosterops lateralis* | Vanuatu | Moyle et al. 2009 | LSUMNS B45835 | FJ460804 | FJ460872 | FJ460941 |
| *Zosterops lateralis* | Australia | Moyle et al. 2009 | KUNHM 6094 | FJ460814 | FJ460882 | FJ460951 |
| *Zosterops fuscicapillus* | Louisiade Is. | Moyle et al. 2009 | NMNH 2003–062 | FJ460829 | FJ460896 | FJ460965 |
| *Zosterops metcalfi* | Choiseul, Solomon Is. | Moyle et al. 2009 | UWBM 63177 | FJ460817 | FJ460885 | FJ460954 |
| *Zosterops stresemanni* | Malaita, Solomon Is. | Moyle et al. 2009 | UWBM 66034 | FJ460819 | FJ460887 | FJ460956 |
| *Zosterops ugiensis* | Makira, Solomon Is. | Moyle et al. 2009 | KUNHM 12803 | FJ460836 | FJ460903 | FJ460972 |
| *Zosterops luteus* | Australia | Moyle et al. 2009 | KUNHM 8904 | FJ460812 | FJ460880 | FJ460949 |
| *Zosterops rendovae* | Rendova, Solomon Is. | Moyle et al. 2009 | UWBM 76258 | FJ460832 | FJ460899 | FJ460968 |
| *Zosterops rendovae* | Tetepare, Solomon Is. | Moyle et al. 2009 | UWBM 76356 | FJ460830 | FJ460897 | FJ460966 |
| *Zosterops kulambangarae* | Kohingo, Solomon Is. | Moyle et al. 2009 | UWBM 76278 | FJ460831 | FJ460898 | FJ460967 |
| *Zosterops splendidus* | Rannonga, Solomon Is. | Moyle et al. 2009 | AMNH DOT171 | FJ460835 | FJ460902 | FJ460971 |
| *Zosterops luteirostris* | Ghizo Is, Solomon Is. | Moyle et al. 2009 | AMNH DOT113 | FJ460834 | FJ460901 | FJ460970 |
| *Zosterops vellalavella* | Vellalavella, Solomon Is. | Moyle et al. 2009 | AMNH DOT166 | FJ460828 | FJ460895 | FJ460964 |
| *Zosterops rennelianus* | Rennel, Solomon Is. | Moyle et al. 2009 | UWBM 69808 | FJ460818 | FJ460886 | FJ460955 |
| *Zosterops griseotinctus* | Louisiade Is. | Moyle et al. 2009 | NMNH 2003–067 | FJ460820 | FJ460888 | FJ460957 |
| *Zosterops murphyi* | Kolombangara, Solomon Is. | Moyle et al. 2009 | AMNH DOT193 | FJ460833 | FJ460900 | FJ460969 |

Footnote

FMNH, The Field Museum of Natural History; KUNHM, University of Kansas Natural History Museum; LSUMNS, Louisiana State University Museum of Natural Science; USNM, National Museum of Natural History; UWBM, University of Washington Burke Museum; AMNH, American Museum of Natural History; CMNH, Cleveland Museum of Natural History
