# Supplementary material for: Non-sister Sri Lankan white-eyes (genus Zosterops) are a result of independent colonizations
Source: PLoS One. 2017 Aug 9;12(8):e0181441. doi: 10.1371/journal.pone.0181441 (PMC5549887; doi:10.1371/journal.pone.0181441)
Supplement: S2 Table — (DOCX) [file pone.0181441.s004.docx]

**S2 Table:**

**Eigen values for each variable in each Principal Component (PC) resulted from the Principal Component Analysis**

**(**Grey coloured boxes and bold text show the highest eigen value contributed to each PC).

|  | **PC1** | **PC2** | **PC3** |
| --- | --- | --- | --- |
| Elevation (m) | 0.36482 | -0.23885 | -0.10005 |
| Head length | 0.28391 | -0.32284 | 0.28539 |
| **Head width** | 0.29422 | 0.05657 | 0.49118 |
| **Total culmen** | 0.40107 | -0.02808 | -0.25555 |
| Exposed culmen | 0.36805 | -0.17884 | -0.35373 |
| Bill height (mm) | 0.30010 | 0.33536 | -0.22823 |
| Bill width (mm) | 0.30403 | 0.34655 | 0.25163 |
| **Eye ring [a] – height of the eyebrow** | 0.07388 | 0.53702 | -0.35079 |
| Eye ring [b] - Eye-ring gap | 0.31511 | -0.06915 | -0.21452 |
| Eye ring [c] - Height of the eye (with eye ring) | 0.20860 | 0.44226 | 0.40661 |
| Flattened wing length | 0.26954 | -0.28534 | 0.16446 |
